# Surgical Management of Spinal Dural Arteriovenous Fistula: A Case Report

**DOI:** 10.7759/cureus.79440

**Published:** 2025-02-22

**Authors:** Kaoutar Faraj, Hamza Sriri, Mohammed Oualid Hmamouch, Hammoud Marouane, Lakhdar Fayçal, Benzagmout Mohammed, Chakour Khalid, Chaoui Faiz Mohammed

**Affiliations:** 1 Department of Neurosurgery, Hôpital Universitaire Hassan II de Fès, Fez, MAR; 2 Department of Neurosurgery, Centre Hospitalier Universitaire (CHU) Hassan II, Fez, MAR

**Keywords:** endovascular treatment, medullary cone edema, spastic myelopathy, spinal dural fistula, spinal flow void, surgery treatment

## Abstract

Spinal dural arteriovenous fistulas (SDAVFs) are the most commonly encountered vascular malformation of the spinal cord and a treatable cause of progressive myelopathy. They most commonly affect elderly men and are classically found in the thoracolumbar region.

The arteriovenous shunt is located inside the dura mater close to the spinal nerve root where the arterial blood from a radiculomeningeal artery enters a radicular vein. The increase in spinal venous pressure leads to the decreased drainage of normal spinal veins, venous congestion, and the clinical findings of progressive myelopathy.

We report the case of a 62-year-old patient who presented with spastic paraparesis 3/5 bilaterally, with thermalogic hypoesthesia, hyperreflexia, and genito-sphincter disorders.

Spinal cord MRI and spinal cord arteriography confirmed the diagnosis of dorsal dural arteriovenous fistula regarding D11, which was excluded by surgical technique, with the disappearance of serpiginous venous dilatations in the perimedullary vein.

A good postoperative clinical evolution has been noted, with a clear improvement in the deficit.

We report the case of a patient with a dorsal medullary dural fistula; we will discuss the clinical and neuroimaging presentation of the dorsal spinal dural fistula and the surgical management.

## Introduction

Spinal dural arteriovenous fistulas (SDAVFs) with perimedullary venous drainage are rare, with an annual incidence of about 5-10 cases per one million people [1]; they are one of the most common vertebro-medullary vascular malformations [2].

They are generally diagnosed in the middle-aged population, especially in men, and are usually located in the thoracolumbar region in 90% of cases, and they are presented as progressive spastic myelopathy with genito-sphincter disorders [3].

Spinal cord MRI allows to show the signs of the spinal dural fistula, but only spinal angiography can confirm the certainty of the diagnosis.

The treatment has two options: surgical and endovascular management.

The prognosis is relatively favorable, requiring early diagnosis and early treatment before the onset of serious neurological deficits [4].

## Case presentation

We report the case of a 62-year-old man, with a past medical history of inguinal hernia and cholecystectomy, who presented to the emergency department with gait disturbances: progressive weakness of both lower extremities and sensitivity loss for five months, evolving in the context of apyrexia and the preservation of the general condition.

On neurological examination, he had spastic paraparesis rated 3/5 bilaterally on the manual muscle test (MMT), with thermalogic hypoesthesia, hyperreflexia (brisk osteotendinous reflexes and epileptoid trepidation), and Babinski's sign bilaterally, as well as genito-sphincter disorders such as erectile dysfunction and urinary incontinence.

**Figure 1:**
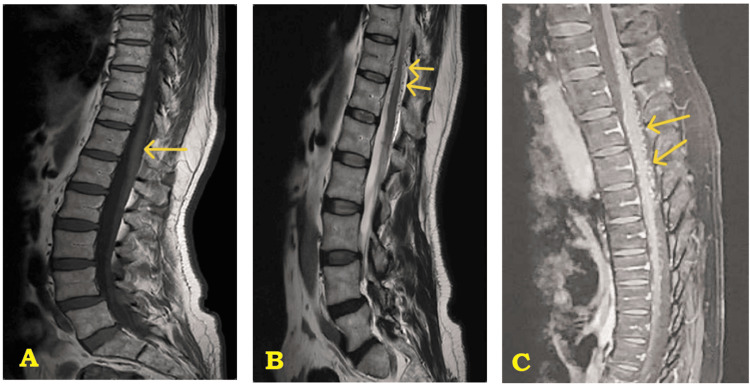
Sagittal thoracolumbar spine MRI demonstrating diffuse long-segment spinal cord signal abnormality (edema of the medullary cone and flow voids). (A) T1-weighted sagittal sequence: intramedullary hypointense signal; medullary cone enlargement: edema (arrow). (B) T2-weighted sagittal sequence: hyperintense signal in the medullary center on T2, with hypointense tortuous structures in the perimedullary vein (arrows: tortuous flow voids). (C) T1 with gadolinium injection-weighted sagittal sequence: hyperintense tortuous structures in the perimedullary vein (arrows: tortuous flow voids).

Spinal cord MRI was done first; it allows to show medullary edema with an enlargement of the spinal cord, which is the site of a centromedullary signal anomaly in the T1 hypointense signal (Figure 1A) and T2 hyperintense signal (Figure 1B), extending from D8 to D12; it refers to myelopathy by venous hyperpressure, with the individualization of multiple serpiginous tortuous structures in the perimedullary vein in T2 hypointense signal, which are enhanced with gadolinium; it refers to dilated perimedullary vascular structures (flow void) (Figure 1C).

Spinal angiography was performed via a transfemoral approach under local anesthesia. It demonstrated the vascular anatomy of the spinal cord with great detail and could identify the shunt between a radiculomeningeal artery and a radiculomedullary vein, subsequently showing the dilatation of the perimedullary venous plexus located along the dorsal surface of the spinal cord.

**Figure 2:**
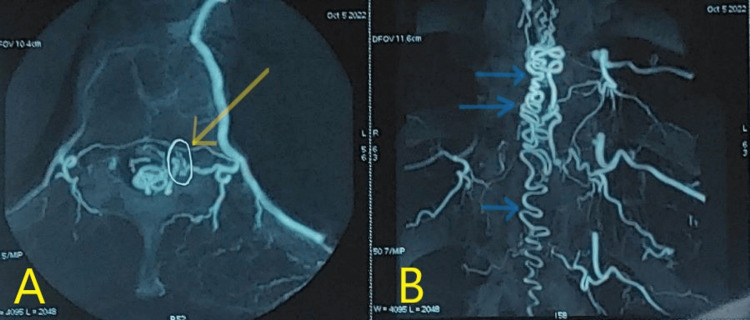
Pretreatment spinal angiography. (A) Axial angiography sequence: the shunt between a radiculomeningeal artery supplied by the 11th intercostal artery with a perimedullary vein in front of the conjugation hole of the left D11 root (yellow arrow). (B) Coronal angiography sequence: tortuous perimedullary veins around the posterior side of the spinal cord (blue arrows).

In our case, it demonstrated the communication between a radiculomeningeal artery supplied by the 11th intercostal artery and a perimedullary vein in front of the root hole of the left D11 root, responsible for the dilation of the perimedullary venous plexus (Figure 2A), thus confirming the presence of a dural arteriovenous shunt in favor of a dorsal medullary dural fistula D11 (Figure 2B).

The embolization of the fistula was attempted but fails to navigate the catheter into the tortuous vessels; then, a surgical exclusion of the dural fistula was performed.

**Figure 3:**
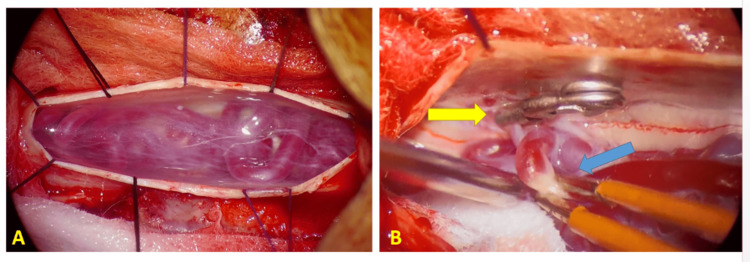
Microscopic intraoperative view. (A) Microscopic intraoperative view after opening the dura mater shows the dilated and tortuous vessels in the posterior side of the spinal cord. (B) Microscopic intraoperative view shows the radiculomeningeal vein clipped temporarily (in the thickness of the dura mater in front of the root hole of the left D11 dorsal root) and then coagulated.

The surgical procedure was performed through a D11 hemilaminectomy, dural opening, and arachnoid band dissection, and then, the vein was identified and clipped temporarily and then coagulated (Figure 3).

**Figure 4:**
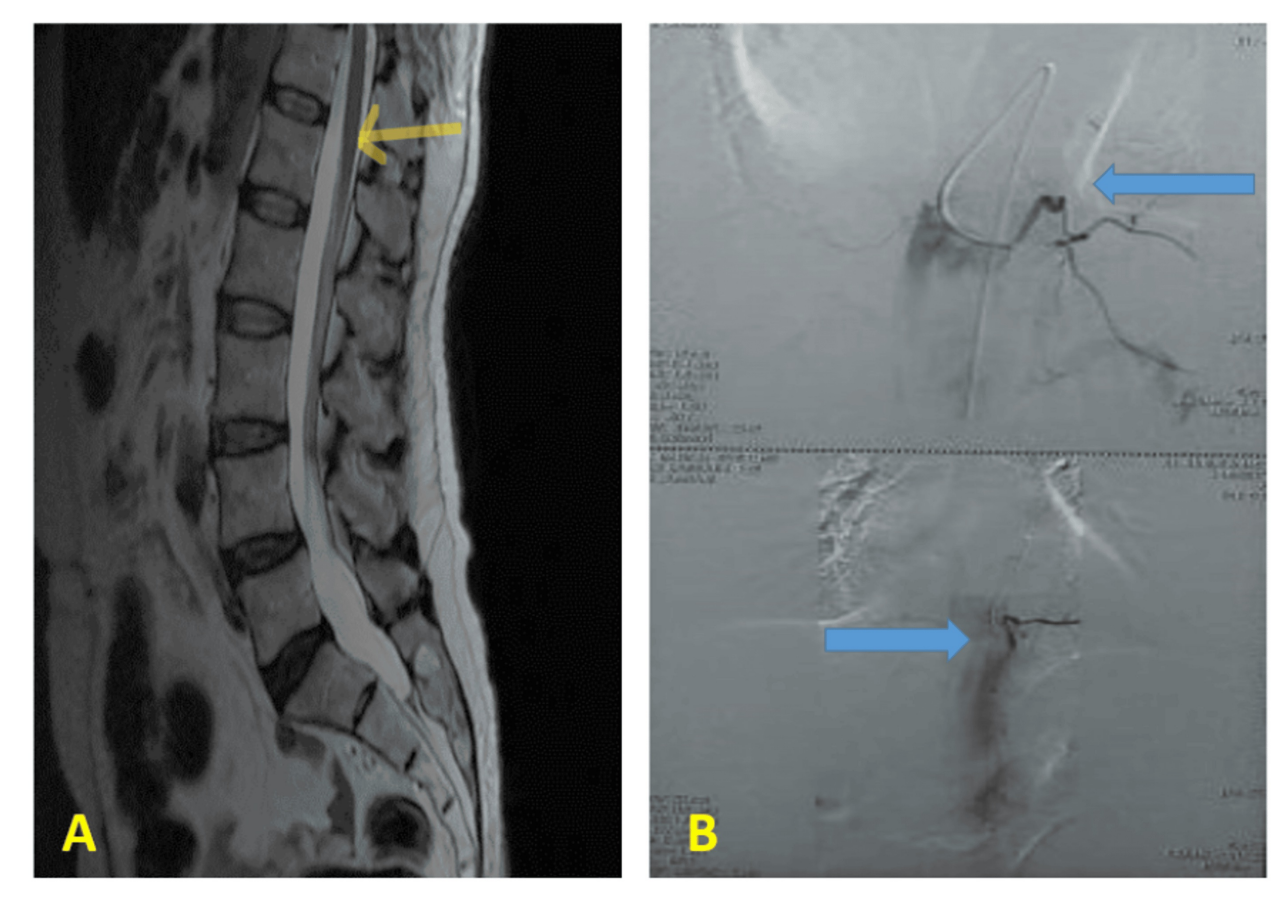
Postoperative spinal MRI and spinal angiography. (A) Postoperative spinal MRI: T2-weighted sagittal sequence control spinal MRI that shows spinal cord hypersignal regression (yellow arrow) and the disappearance of flow void around the spinal cord. (B) Postoperative spinal angiography shows the complete exclusion of the dural fistula and the disappearance of serpiginous venous dilatations in the perimedullary vein (blue arrows).

A spinal cord MRI (Figure 4A) and a spinal cord angiography (Figure 4B) were performed; they demonstrate the complete exclusion of the dural fistula, as well as the disappearance of serpiginous venous dilatations in the perimedullary vein.

A good postoperative clinical evolution was noted, with a clear improvement in paraparesis with the complete recovery of muscle strength, rated 5/5 on neurological examination, and the patient was able to resume his activities, in two months postoperatively.

## Discussion

Spinal dural fistulas are defined as a microscopic arteriovenous shunt of 40-140 µm, located in the intramedullary space, and are usually located in the thickness of the dura mater, in front of the intervertebral conjugation foramen; they represent type I according to Spetzler's classification [5].

It predominates in men in the fifth decade; their topography is essentially dorsal and dorsolumbar, and rare cases of cervical or sacral localization have been reported.

It consists of a direct connection between one or more radiculomeningeal arterioles and a single root vein draining to the perimedullary veins with a chronic increase in venous pressure, which is responsible for the vasodilation of the intramedullary veins and intramedullary venous congestion that predominates at the level of the medullary cone; this congestion leads to chronic hypoxia and progressive myelopathy [6].

The clinical manifestations of venous congestion tend to be insidious and non-specific, which is why the diagnostic delay is often greater than one year; it constitutes progressive myelopathy associated with motor disorders affecting the lower limbs, most often accompanied by sensory and sphincter disorders such as erectile dysfunction and bladder incontinence [7,8].

Spinal cord MRI is performed in the first-line examination when SDAVFs are suspected. The examination must include a whole spine examination with T1 and T2 sequence exploration in all three planes and include injection of gadolinium; the fistula itself is not visible being of microscopic size; the MRI allows to show the signs of the spinal dural fistula, which are the enlargement of the medullary cone (constant but non-specific sign), abnormalities of the intramedullary signal such as an intramedullary T2 hyperintense signal and a T1 hypointense signal with enhancement after gadolinium injection, and perimedullary signal abnormalities such as serpiginous signal void structures hypointense on the T1 and T2 sequences and enhancing after gadolinium injection, corresponding to dilated perimedullary drainage veins [9].

Spinal angiography, considered a "gold standard," provides the angioarchitecture of the spinal cord and remains the only examination that can confirm the certainty of the diagnosis and the location of SDAVFs; it allows the visualization of the shunt, the nourishing pedicles, and the drainage veins [2].

The management of SDAVFs includes two options; it can be done either surgically or endovascularly and consists of interrupting the shunt between radiculomeningeal arterioles and the medullary vein.

The surgical treatment of spinal dural arteriovenous fistulas was performed for the first time in 1916 by Elsberg [10]. It is based on the coagulation of the drainage vein, thus disconnecting the fistula from the spinal veins; this surgical procedure is relatively safe and simple and is performed through a laminectomy limited to the fistula floor, with the opening of the dura mater and the identification of the efferent root vein to the fistula and then its coagulation [11].

The surgical exclusion of the fistula is a definitive and curative technique [12,13]; according to previous studies, the success rate of surgical treatment is higher than endovascular treatment, with a low complication rate [14,15]. Steinmetz et al. [16] recommended surgical treatment as first-line treatment, due to its safety, efficacy, and improvement in neurological symptoms in most patients; it provides a successful occlusion rate (98%), with 2% morbidity and no mortality [17,18].

It is recommended as first-line treatment if the selective introduction of the microcatheter is difficult, after failed embolization, and after recurrence with embolization and also if the arteries that supply the fistula originate from the arteries that contribute to the vascularization of the spinal cord [19]; however, endovascular treatment can be proposed in some selected cases, such as elderly patients with comorbidities who could run greater risks undergoing surgery.

The embolization of a spinal dural arteriovenous fistula was performed for the first time by Doppman et al. in 1968 [20]; actually, many lesions can be the subject of endovascular intervention due to the improvement of embolic agents. Particle embolization is not recommended in the case of fistula due to its high rates of recanalization; that is why it is recommended to use liquid embolization to prevent recanalization [21,22].

Currently, endovascular treatment has been improved [16]. It has a low morbidity rate and a high chance of recovery. With a success rate varying between 70% and 89.5%, despite advances in endovascular techniques, surgery is still the most definitive technique with the highest rate of complete occlusion, but the effectiveness and overall durability of endovascular treatment are still inferior to surgical occlusion [22].

SDAVFs are considered a treatable cause of myelopathy, so it is crucial to treat it quickly once the diagnosis has been made because the neurological prognosis remains closely related to the severity of myelopathy and the duration of symptoms before treatment.

A spinal angiography after fistula disconnection is crucial to look for the disappearance of the dural arteriovenous shunt, and the MRI signal abnormalities will gradually disappear; intramedullary T2 hyperintense signal and vascular abnormalities regress from the first month after treatment, and the enlargement of the medullary cone can evolve into atrophy [23,24].

Approximately two-thirds of patients would have an improvement in motor deficit and an improvement in their sensory disorders in a third of cases, but genito-sphincter disorders are reversible in variable degrees.

## Conclusions

Spinal dural arteriovenous fistula is a rare but disabling condition and is considered the only treatable cause of myelopathy. Any progressive myelopathy in a patient over 40 years old should prompt suspicion of this diagnosis and lead to the rapid performance of a spinal MRI, supplemented by spinal angiography for definitive confirmation if suggestive signs are present. Early diagnosis and prompt treatment are recommended before the onset of severe neurological deficits, and a clear understanding of the malformation's angioarchitecture is essential for effective disconnection.
